# Measuring ketone bodies for the monitoring of pathologic and therapeutic ketosis

**DOI:** 10.1002/osp4.516

**Published:** 2021-05-04

**Authors:** Joseph C. Anderson, Samer G. Mattar, Frank L. Greenway, Richard J. Lindquist

**Affiliations:** ^1^ Department of Bioengineering University of Washington Seattle Washington USA; ^2^ Department of Surgery Baylor College of Medicine Houston Texas USA; ^3^ Pennington Biomedical Research Center Baton Rouge Louisiana USA; ^4^ Department of Family Medicine Swedish Medical Center Seattle Washington USA

**Keywords:** β‐hydroxybutyrate, acetone, bariatric surgery, metabolism

## Abstract

**Background:**

The ketone bodies β‐hydroxybutyrate (BOHB) and acetone are generated as a byproduct of the fat metabolism process. In healthy individuals, ketone body levels are ∼0.1 mM for BOHB and ∼1 part per million for breath acetone (BrAce). These levels can increase dramatically as a consequence of a disease process or when used therapeutically for disease treatment. For example, increased ketone body concentration during weight loss is an indication of elevated fat metabolism. Ketone body measurement is relatively inexpensive and can provide metabolic insights to help guide disease management and optimize weight loss.

**Methods:**

This review of the literature provides metabolic mechanisms and typical concentration ranges of ketone bodies, which can give new insights into these conditions and rationale for measuring ketone bodies.

**Results:**

Diseases such as heart failure and ketoacidosis can affect caloric intake and macronutrient management, which can elevate BOHB 30‐fold and BrAce 1000‐fold. Other diseases associated with obesity, such as brain dysfunction, cancer, and diabetes, may cause dysfunction because of an inability to use glucose, excessive reliance on glucose, or poor insulin signaling. Elevating ketone body concentrations (e.g., nutritional ketosis) may improve these conditions by forcing utilization of ketone bodies, in place of glucose, for fuel. During weight loss, monitoring ketone body concentration can demonstrate program compliance and can be used to optimize the weight‐loss plan.

**Conclusions:**

The role of ketone bodies in states of pathologic and therapeutic ketosis indicates that accurate measurement and monitoring of BOHB or BrAce will likely improve disease management. Bariatric surgery is examined as a case study for monitoring both types of ketosis.

## 1 INTRODUCTION

The recent popularity of the ketogenic diet (KD) has kindled a renewed interest in the creation and metabolism of ketone bodies and the state of ketosis. This diet, characterized by high‐fat and very low‐carbohydrate (<50 g/day) intake, appears to result in improvements in weight management, metabolic syndrome, and cognition.^1^, ^2^, ^3^, ^4^ Implicitly, the diet mandates a marked reduction in carbohydrate intake, which results in the generation and utilization of ketone bodies, instead of carbohydrates, to fuel the brain and other neurologic tissues. Many authorities assumed that ketone bodies, in states of carbohydrate restriction, are the primary drivers for the perceived benefits of the KD. Because of this success, an explosion in research has taken place to identify additional interventions that may increase ketosis (e.g., intermittent fasting and exogenous supplementation) and to better understand the effects of ketosis in health and disease.^5^, ^6^, ^7^, ^8^

**FIGURE 1:**
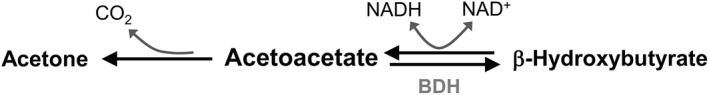
Acetoacetate, formed primarily from β‐oxidation of fatty acids, can be reduced to β‐hydroxybutyrate (BOHB) or decarboxylated to acetone. Beta‐hydroxybutyrate dehydrogenase (BDH) interconverts acetoacetate and BOHB depending on intercellular conditions (e.g., NADH). Acetone is produced via spontaneous or catalytic decarboxylation of acetoacetate. NADH, nicotinamide adenine dinucleotide

Ketosis is defined as the elevated concentration of ketone bodies in the blood. Ketone bodies are comprised of three chemicals: acetoacetate, β‐hydroxybutyrate (BOHB), and acetone. Acetoacetate is created in the liver from free fatty acids (FFAs) when glucose availability is limited. Acetoacetate can be enzymatically interconverted into BOHB.^9^ Additionally, acetoacetate can be decarboxylated, spontaneously or by catalytic action, into acetone (Figure 1).^10^ Ketone body concentrations increase with corresponding increases in fat metabolism.^11^, ^12^

Recent studies have also demonstrated a number of non‐diet‐related disease conditions that can cause elevated ketosis.^13^, ^14^ These diseases cause hyperketonemia for a variety of reasons, including poor insulin signaling in diabetes, impaired fatty acid metabolism in heart failure (HF), and slow ketone body elimination as a result of genetic disorders. It is logical, therefore, that monitoring ketosis levels may provide a complete picture of the behavior and severity of the underlying disease. Additionally, periodic ketosis monitoring may help in the management and treatment of the disease.

Some diseases appear to respond favorably to an increase in ketosis. It has been demonstrated that once ketones reach “therapeutic” levels, they help attenuate disease severity or result in disease regression.^15^, ^16^, ^17^ Examples include neurologic diseases where the ketone bodies provide fuel to metabolically compromised brain regions and treatment of type 2 diabetes to improve diabetic sequelae and reduce medications. Thus, monitoring ketosis levels can help maintain therapeutic concentrations of ketone bodies to optimize disease treatment.

Additionally, weight loss creates an elevation in fat metabolism that is reflected in elevated ketone body concentrations.^11^, ^18^ Elevated fat metabolism is correlated with increased ketone body concentrations.^19^ Thus, medical weight loss and weight loss prior to bariatric surgery may be optimized and compliance assessed by monitoring ketone bodies as surrogate markers of subject‐specific fat metabolism.

Measurement of ketone bodies is becoming more common because of an increase in the number of relatively inexpensive consumer devices. These chemical sensors typically measure acetoacetate in urine, BOHB in blood, or acetone in breath (BrAce). While urine samples are common, urine acetoacetate is not typically assessed using a quantitative method causing significant measurement uncertainty.^20^, ^21^ Currently, blood BOHB is the gold standard for assessing ketosis. However, the measurement of BrAce is becoming more widely accepted as a reliable indicator, particularly at low ketone body levels where BrAce has greater sensitivity to change than blood BOHB.^19^ Ketone body concentrations for two reference states are as follows (Table 1): (1) healthy individuals on a balanced macronutrient diet typically have ketone body concentrations of BOHB ∼0.1 mM or BrAce ∼1 part per million (ppm); and (2) subjects in nutritional ketosis (i.e., keto‐adaptation) have ketone body concentrations of at least BOHB = 0.5 mM^35^, ^41^, ^42^ or BrAce ≥ 9 ppm.^19^, ^22^

**TABLE 1:** Ketone body (BOHB in mM or BrAce in ppm) concentration ranges observed in health, for disease states and for therapeutic benefit (minimum concentration thresholds)

Ketosis	Condition	Range or threshold		References
		BOHB (mM)	BrAce (ppm)	
Health	Balanced macronutrient diet	0.1	1	^19^
	Nutritional ketosis	0.5	9	^19^, ^22^
Disease	Heart failure	>0.2	2–20	^23^, ^24^, ^25^, ^26^
	Ketoacidosis	>3.0	>75	^9^, ^13^, ^14^, ^19^, ^27^, ^28^
	Genetic disorders	―	―	See text
Therapy	Brain function: Alzheimer’s	0.5	9^a^	^29^, ^30^
	Brain function: Parkinson’s	1.0	―	^31^
	Brain function: Dementia	0.5	9^a^	^32^
	Brain function: Migraine	4.0	―	See text
	Cancer	0.5	9^a^	^33^, ^34^
	Type 2 diabetes	0.5	9^a^	^35^, ^36^
	Epilepsy	4.0	―	^37^, ^38^, ^39^, ^40^
	Weight loss	―	2	^11^
Bariatric surgery	Ketoacidosis	>3.0	>75	See text and references above
	Weight loss	―	2	

Abbreviations: BOHB, β‐hydroxybutyrate; BrAce, breath acetone.

^a^ Nutritional ketosis. BrAce ≥ 9 ppm when BOHB = 0.5 mM.^19^, ^22^

The concentrations of ketone bodies observed for a range of diseases haven't been summarized. This review describes diseases that cause ketosis (ketogenic diseases), their underlying ketogenic mechanisms, and the ranges of ketosis (as defined by blood BOHB and BrAce concentrations) that are associated with the disease. For diseases that can be treated with an elevation in ketosis (therapeutic ketosis), the underlying mechanism of therapeutic ketosis and the thresholds of ketone body concentrations associated with a therapeutic benefit are detailed. Measurement of ketone body concentrations is needed to demonstrate the achievement of a therapeutic ketone body level. Additionally, ketone body elevation during weight loss and how it can be used to optimize fat loss are reviewed. Finally, a case study on bariatric surgery provides a vignette for how monitoring ketone bodies can be used for disease and therapeutic benefit.

## 2 KETOSIS FROM DISEASE

### 2.1 Heart failure

In health, fatty acids provide 50%–70% of the heart's energy.^43^ In HF, myocardial fatty acid metabolism is impaired which may be due to a downregulation of myocardial proteins used to metabolize fatty acids. However, lipolysis and FFAs are elevated in HF because of increases in stress hormones (e.g., cortisol), cytokines, malnutrition, or cardiac cachexia.^23^, ^24^, ^44^, ^45^ Unable to metabolize FFA, the failing heart shifts to other fuels including ketone bodies which are elevated in HF.^46^ Greater concentrations of ketone bodies are observed because (1) the abundance of serum FFA causes a rise in liver ketogenesis, the source of ketone bodies; and (2) skeletal muscles have lower consumption of BOHB.^44^, ^46^ BOHB can be rapidly utilized by the metabolically compromised myocardium because the enzymes required for ketone body metabolism are more abundant in HF.^43^, ^44^

In scientific studies, subjects with HF have 2–20‐fold greater levels of the ketone body acetone in breath (BrAce, Table 1) as compared to healthy controls (BrAce ∼ 1 ppm) or to cardiac patients without HF.^23^, ^25^, ^26^ Increases (decreases) in BrAce correspond to increased (decreased) HF severity.^23^, ^24^, ^47^ Thus, monitoring BrAC may provide a marker of HF deterioration or improvement.^24^, ^48^

### 2.2 Ketoacidosis

In healthy individuals, insulin in the blood interacts with the cell membrane to facilitate the uptake of blood glucose by the cell. Additionally, the interaction of insulin with adipose tissue suppresses lipolysis. In individuals with diabetes, cells are unable to receive the insulin signal because either insulin is not present (type 1 diabetes) or the cell is insensitive to insulin and doesn't respond to its presence (type 2 diabetes). Thus, glucose is not taken up by the cell, and the concentration of glucose in the blood rises. Without the insulin signal, lipolysis is no longer inhibited, plasma FFA rises, and hepatic ketone body production increases.^49^ In lieu of glucose as a primary fuel source, cells can use fatty acids and ketone bodies to meet their energy requirements. Without the insulin signal to reduce blood sugar and suppress fat breakdown, glucose (hyperglycemia), ketone bodies (hyperketonemia), and hydrogen ions (acidosis) can increase dramatically in the blood and, without intervention, lead to diabetic ketoacidosis (DKA).^27^, ^50^

Because ketone bodies are a precursor and marker of DKA, measurement of ketone bodies can help identify the development, assess the severity, and assist in monitoring the resolution of DKA. As ketoacidosis develops, BOHB and BrAce increase from healthy levels (BOHB ∼ 0.1 mM or BrAce ∼1 ppm) to those associated with the onset of ketoacidosis (BOHB > 3 mM or BrAce > 75 ppm) (Table 1). Because of the large concentration range between healthy ketone body levels and ketoacidosis, ketone body monitoring can provide a critical tool to alert providers and patients that a healthy ketosis state is developing into ketoacidosis, allowing expedient treatment before it reaches a critical level.^28^, ^51^ When DKA is present, measurement of BOHB and BrAce may help to assess the severity of ketoacidosis. BOHB (3–20 mM) and BrAce (75–1250 ppm) range considerably in people with DKA, and the magnitude of these ketone bodies may be associated with increased severity of DKA.^19^, ^52^ After the intervention, scientific studies have demonstrated that ketone body measurement can improve the course of DKA resolution.^53^, ^54^ Four diabetes associations recommend ketone body monitoring, typically BOHB, during DKA resolution.^55^ During DKA, the concentration of BOHB is much greater than acetoacetate.^56^ As DKA is treated, BOHB decreases by conversion to acetoacetate, which causes an elevation of acetoacetate. Because DKA is not resolved until both BOHB and acetoacetate concentrations return to baseline, monitoring BrAce, a product of acetoacetate decarboxylation, at 30‐min intervals, can demonstrate the full resolution of DKA.^27^, ^53^ Additionally, monitoring ketone bodies during resolution should reduce the duration and cost of medical treatment.^57^

Prevention of DKA may be the best use of ketone body measurement. DKA requires immediate medical attention (e.g., emergency room visit), with an average cost for hospitalization of $26,566 in 2014.^58^ To reduce DKA events and hospitalization, scientific studies recommend self‐monitoring of ketone bodies in patients with type 1 diabetes, insulin‐dependent type 2 diabetes, sustained blood glucose concentration >300 mg/dl, acute illness, or stress.^20^, ^27^, ^56^ Patients have indicated that measurements of elevated BOHB were useful in determining subsequent insulin dose and food intake.^59^ During sick days, particularly those involving nausea, vomiting, or infections, scientific studies recommend monitoring BOHB throughout the day, specifically for young children because of the frequency of illness in this population.^59^ Studies indicate that children and adults who measure BOHB during “sick days” can prevent the onset of DKA, reduce the time to DKA resolution, reduce monetary costs, and decrease the rate of hospitalizations.^55^, ^59^, ^60^

### 2.3 Euglycemic ketoacidosis

In addition to ketoacidosis associated with poorly controlled diabetes, ketoacidosis can occur in patients with diabetes who control their blood sugar with a sodium–glucose cotransporter‐2 (SGLT2) inhibitor. The SGLT2 inhibitor eliminates excess blood glucose through excretion by the kidneys and may lead to ketoacidosis, in a subset of individuals with diabetes, even though blood glucose is well controlled (i.e., euglycemic ketoacidosis).^61^, ^62^, ^63^, ^64^

This condition appears to be driven by low insulin levels in individuals with impaired insulin secretion, poor fluid intake, low carbohydrate intake, and/or fasting.^51^, ^62^, ^65^ Because the SGLT2 inhibitor maintains blood glucose within a “healthy range”, blood glucose monitoring will not alert individuals or clinicians to the developing ketoacidosis. Thus, measurement of ketone bodies is important for these subjects, perhaps even more so on sick days. The monitoring guidelines outlined for ketoacidosis (above) could be applied. The large concentration differential between healthy ketone body levels and ketoacidosis can be exploited to monitor elevations in ketosis. Significant elevations can be addressed before a crisis develops.^28^, ^51^

Interestingly, pregnant women with diabetes (<3% of all diabetic gestations) can have euglycemic ketoacidosis which may progress more rapidly as compared to nonpregnancy states.^66^ Thus, ketone body measurement may identify the early stages of hyperketonemia before it escalates to DKA.

### 2.4 Genetic disorders

Genetic disorders can elevate ketosis through the overproduction of ketone bodies or impairment of ketone body utilization. Hepatic ketone body production elevates when low blood glucose causes a reduction in insulin and an increase in circulating fatty acids. Normal glucose levels, maintained via glycogen metabolism or gluconeogenesis, are dependent on key enzymes such as glycogen synthase, glycogen phosphorylase kinase, fructose‐1,6‐diphosphatase, or glucose‐6‐phosphatase. Genetic disorders can prevent the expression of these enzymes, which would cause fasting hypoglycemia and accelerated ketogenesis leading to hyperketonemia.^14^

**FIGURE 2:**
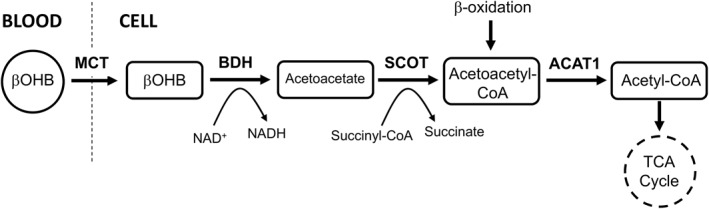
Pathway for utilization of ketone bodies (ketolysis) where deficiencies in SCOT or ACAT1 cause significant ketonemia (adapted from Aubert et al.^67^). β‐oxidation is output from β‐oxidation of fatty acids. ACAT1, acetyl‐CoA acetyltransferase1; BDH, β‐hydroxybutyrate dehydrogenase; MCT, monocarboxylate transporter; SCOT, succinyl‐CoA:3‐oxoacid‐CoA transferase

A lack of peripheral tissue utilization will cause ketone body elevation. The pathway for ketolysis is controlled by two enzymes (Figure 2): succinyl‐CoA:3‐oxoacid‐CoA transferase (SCOT) and acetyl‐CoA acetyltransferase1 (ACAT1), which early literature identified as 2‐methylacetoacetyl‐CoA thiolase (MAT). A lack of these enzymes causes hyperketonemia and ketoacidosis, particularly in a fasting state.^68^, ^69^

Monitoring ketonemia via BOHB or BrAce measurement can provide a quantitative check on dietary compliance, particularly on sick days, and a method to minimize the conversion of hyperketonemia to ketoacidosis. Ketone body thresholds have not been described but would likely be similar to those for ketoacidosis (BOHB > 3 mM or BrAce > 75 ppm—see Section 2.2).

## 3 KETOSIS FOR THERAPY

In addition to weight loss, multiple obesity‐associated diseases respond favorably to elevated ketosis. These diseases appear to have impaired glucose metabolism (e.g., brain dysfunction) or an inability to manage elevated insulin and glucose (e.g., cancer, diabetes). Elevated BOHB may provide relief by replacing glucose as a primary source of energy and removing oxidant species.^15^, ^16^ For these obesity‐associated diseases, carbohydrate restriction may be the optimal modality for elevating ketone bodies while decreasing insulin and blood glucose concentration. Therapeutic BOHB concentrations are primarily disease dependent and modulated by a multitude of factors including age, gender, weight, diet, and disease severity. Thus, sequential ketone body measurements over time can demonstrate that the threshold for therapeutic ketosis has been achieved and maintained.

### 3.1 Brain function

In health, glucose fuels the brain. During starvation, glucose is scarce and FFAs are abundant. Because the brain cannot use fat for fuel, the body converts fat into ketone bodies to fuel the brain.^52^, ^70^ Scientific studies have demonstrated that elevated ketone bodies (via KD, supplementation with medium chain triglyceride, fasting, etc.) can improve brain function in subjects with Alzheimer's, dementia, and Parkinson's disease.

It appears that these diseases are characterized by the inability of specific brain regions to use glucose for fuel, which causes regional dysfunction in the brain. When available, ketone bodies can fuel diseased brain regions resulting in improvement of neurologic function. At BOHB concentrations >4 mM, ketone bodies are estimated to supply more than 50% of the brain's energy requirement.^71^

#### 3.1.1 Alzheimer's

Many factors drive Alzheimer's disease including insulin resistance, genetic defects, and a regional reduction in glucose metabolism which is correlated to decrements in cognitive scores.^7^, ^72^ Elevation in BOHB to ∼0.5 mM (BrAce ≥ 9 ppm) via consumption of ketogenic foods appears to improve cognitive function.^29^, ^30^, ^73^, ^74^ Additionally, improvement in cognitive function is associated with increased BOHB concentrations.^29^, ^30^, ^75^

#### 3.1.2 Parkinson's

The benefits of elevated ketone bodies on mitochondrial activity have been proposed for Parkinson's disease. While similar to the mechanisms for other brain maladies, it is hypothesized that BOHB increases energy production because BOHB may bypass a defect in complex I of the electron transport chain.^76^ In one study, subjects with Parkinson's on a 4‐week KD showed improvement on the Unified Parkinson's Disease Rating Scale. A pilot study showed some symptom resolution when BOHB = 1.0 mM.^31^ While additional studies are needed, it is expected that BOHB must range between 2 and 7 mM to provide a therapeutic effect.^7^, ^77^

#### 3.1.3 Dementia

Older subjects with mild cognitive impairment may experience improved verbal memory performance using a very low carbohydrate diet (<35 g/day on average). Improved memory performance correlated with increases in ketone levels and reductions in insulin levels.^32^ Healthy geriatric individuals will also likely benefit from the best improvement found in subjects with strong dietary compliance.^78^ Based on the dietary criteria, subjects with cognitive impairment may need BOHB concentrations >0.5 mM (BrAce ≥ 9 ppm) to achieve a benefit.^32^

#### 3.1.4 Migraine

Migraine can be characterized as a neurologic inflammation and a reduction in brain metabolism.^79^ To prevent or protect against migraines, elevated ketone bodies may reduce neuroinflammation, inhibit oxidative stress, and modulate mitochondrial function.^80^ Ketotherapeutic benefits for migraines have been known for almost 100 years.^81^ In recent studies, consumption of a very low carbohydrate (<30 g/day) and low‐calorie KD was associated with significant reductions in the number of migraine attacks per month (76% drop) and the number of days with headaches (82% drop).^80^, ^82^ One subject had complete remission of migraine headaches.^83^ Relief was observed within a few days of diet initiation.^79^ Because the diets used are similar to those used for epilepsy therapy, a BOHB greater than 4 mM may be required (Table 1).

#### 3.1.5 Epilepsy

Some subjects with epilepsy have intractable seizures, which are unresponsive to antiepileptic drugs.^84^, ^85^ The frequency of these seizures can, in many cases, be reduced with a KD. In children, approximately 50% of subjects experienced an improvement in seizure frequency when adhering to this diet for a few months.^86^ For children adhering to the diet for 1 year, almost 50% of subjects reported a nearly complete (≥90%) reduction in seizures; similar outcomes persisted for years after study termination.^87^ Adults with epilepsy also experienced a reduction in seizure frequency when placed on KDs. It is clear that elevations in ketone bodies correspond to successful ketogenic therapy.^19^ Scientific studies have shown a relationship between elevated BOHB and improved seizure control in children.^37^, ^38^, ^39^

Ketone body measurement can demonstrate dietary compliance required for therapeutic benefit.^38^, ^88^, ^89^ The therapeutic benefit appears to be around 4 mM for BOHB^37^, ^38^, ^39^, ^40^ (Table 1). When adverse effects of the diet occur (e.g., constipation, bloating, or irregular menstrual cycles), ketone body measurement can provide positive reinforcement, provide information to optimize dietary composition, and hasten return to therapeutic ketosis after a “cheat” day.

### 3.2 Cancer

Rapid and uncontrolled cell growth in cancer is fueled by glucose, an observation named the Warberg effect.^90^ Thus, restricting circulating glucose (e.g., by consumption of a very low‐carbohydrate and high‐fat diet) should cause tumor cells to starve and die.^90^ In healthy cells, energy is produced via aerobic respiration of glucose, fats, or ketone bodies which requires healthy mitochondria, an intact tricarboxylic acid cycle, and a functional electron transport chain. In cancer cells, compromised aerobic respiration forces cancer cells to rely solely on anaerobic glycolysis and aerobic fermentation of glucose for energy.^91^ High corresponding insulin levels signal the expression of glucose transporters (e.g., GLUT3) and glycolytic enzymes.

Carbohydrate restriction will slow cancer growth in three ways. Without fuel, these cells starve and become more susceptible to chemotherapeutics.^91^, ^92^ A decrease in circulating insulin inhibits the signal to upregulate the glucose transporters (e.g., GLUT3) and glycolytic enzymes required for metabolism. Increased BOHB reduces oxidative stress and inflammation, induces apoptosis, and is associated with regression of cancer growth.^33^, ^92^, ^93^, ^94^ Meanwhile, healthy cells thrive via aerobic metabolization of fats and ketone bodies.^90^, ^91^

The few studies using ketotherapy have shown potential benefits (e.g., tumor stability or regression) to the brain (i.e., glioblastoma), breast, lung, and colon cancer.^33^, ^34^, ^93^, ^95^ This regimen likely requires carbohydrate restriction combined with one or more of the following: high‐fat consumption, caloric restriction, fasting, or exogenous ketone supplementation. Measurement of ketosis in cancer patients may help monitor compliance, facilitate dietary modifications, and achieve therapeutic levels of ketone bodies. The minimum threshold for therapeutic benefit appears to be 0.5 mM (BrAce ≥ 9 ppm) and likely depends on the cancer type.^33^, ^34^, ^95^

### 3.3 Type 2 diabetes

One hallmark of diabetes is elevated blood glucose. In people with type 2 diabetes, tissues are insensitive to insulin, resulting in the rise of blood glucose due to increased liver production and a lack of uptake. To achieve normal levels of blood glucose, clinical investigators have proposed “prescribing” a KD to restrict dietary carbohydrates to <50 g/day.^4^, ^96^ After adaption to KD, subjects, on average, experience an improvement in diabetic sequelae and medications, including weight reduction, reduced exogenous insulin, improved insulin sensitivity, and reduced HbA1c.^35^, ^36^, ^96^, ^97^

To achieve these results, clinical studies have measured BOHB levels to verify carbohydrate restriction, to guide the reduction of diabetes medication, and to adjust dietary therapy.^35^, ^36^ Subjects strive to maintain a state of nutritional ketosis, defined as BOHB ≥ 0.5 mM.^35^, ^36^ As an alternative to BOHB, a BrAce ≥ 9 ppm, which corresponded to BOHB ≥ 0.5 mM for 95% of measurements, could be used to demonstrate nutritional ketosis.^19^, ^22^ Daily ketone body measurements demonstrate dietary and lifestyle compliance and may provide a rationale for weaning patients from diabetic medications.^35^, ^36^, ^98^ As lifestyle and dietary factors change with time, ketone body measurements will reflect changes in carbohydrate restriction and, thus, can be used to help compensate for these changes.

### 3.4 Weight loss

During calorie restriction, energy needs are met by mobilizing fat from adipose cells. A portion of the circulating fatty acids is converted into ketone bodies within the liver. The amount of ketone bodies produced is proportional to the rate of fatty acid metabolism within the liver. For subjects on a calorie‐restricted diet, the BrAce concentration has been shown to be proportional to the rate of fat loss.^19^ While BrAce is ∼1 ppm for a typical subject, individuals who lost one‐half pound of fat mass per week on a calorie‐restricted diet had BrAce = 2 ppm. Further elevations in BrAce correlated linearly with increases in fat mass loss.^11^

As a result of the relationship between BrAce and fat mass loss, frequent monitoring of BrAce can be used as a tool by individuals and clinicians to optimize fat loss. During calorie restriction, BrAce ≥ 2 ppm indicates an elevated state of fat metabolism and predicts fat loss if these levels can be maintained. Frequent monitoring provides individuals with immediate feedback to understand how their wellness choices (e.g., diet, exercise, sleep, stress, etc.) affect their state of fat metabolism. Using this feedback, individuals can adjust their choices, daily if needed, to optimize and maintain fat loss and increase compliance (unpublished observations). Coaches and clinicians can utilize longitudinal BrAce measurements to understand individual fat metabolism, to customize the program for each individual, and to counsel patients on how to overcome weight‐loss obstacles. Additionally, BrAce can be used in weight‐loss strategies when preparing for bariatric surgery (discussed below).

One case for measuring BrAce could be tailoring the macronutrient composition to optimize weight loss. In general, customization is difficult because the optimal macronutrient composition for weight loss is not clear. Scientific debates between proponents of a high carbohydrate diet^99^ and supporters of a low carbohydrate diet^100^ have not provided resolution. Optimal diet composition is likely subject dependent. A recent study^101^ suggests a high carbohydrate diet (60% carbohydrate, 20% fat, and 20% protein) is preferred for women with obesity who were insulin sensitive (fasting insulin <10 µU/ml) while a lower carbohydrate diet (40% carbohydrate, 40% fat and 20% protein) is preferred for women with obesity who were insulin resistant. For all subjects, the preferred macronutrient composition gave a ∼twofold increase in body weight loss as compared to the alternative. These findings would indicate that the ability (inability) to efficiently metabolize carbohydrates, as predicted by insulin sensitivity (resistance), may predict the best macronutrient composition for weight loss.

As weight is lost, the body becomes more insulin sensitive. Thus, at some point during weight loss, the best diet may change from a low to a high carbohydrate diet. This change could be monitored with measurements of fasting insulin. However, the cost and invasive nature make this measurement impractical. A different inflection point occurs for others during the first 6 months of dieting. Often when people reach a plateau in their weight loss, they give up dieting and regain the weight.

Measuring BrAce over days and weeks can give feedback on metabolic changes within the patient. A reduction in BrAce from above 2 ppm to near 1 ppm indicates a loss of fat oxidation and, when combined with a weight‐loss plateau, may suggest the need for a dietary change. One cause of this plateau could be the increase in insulin sensitivity which can be quantified via measurement of fasting insulin. If insulin sensitivity has improved, an increase in the carbohydrate content could restart weight loss. Thus, regular monitoring of BrAce during a weight‐loss program could potentially enable personalization of dietary carbohydrates and optimize weight loss over the weight‐loss journey.

### 3.5 Bariatric surgery

Bariatric surgery serves as a case study for monitoring ketone bodies for both pathologic (i.e., ketoacidosis) and therapeutic (i.e., weight loss) ketosis. Over the past half‐century, bariatric surgery has emerged as a valid and effective treatment for significant weight loss and improvement, if not resolution, of associated comorbidities, including diabetes, hypertension, and sleep apnea.^102^ Most of these operations, typically indicated for patients with a BMI > 35, result in the correction of the metabolic dysfunction at the center of the metabolic syndrome and morbid obesity. The dramatic reduction in hunger and the normalization of disturbed metabolic processes result in significant weight loss that is derived from a reduction in both fat and fat‐free mass.^103^, ^104^

Prior to bariatric surgery, weight loss can reduce surgical complications, surgery time, and length of hospital stay.^105^ Very low‐calorie diets (VLCD) are commonly used prior to bariatric surgery as a means to both reduce initial weight and to reduce liver fat mass.^106^ Although VLCDs vary somewhat in calorie and nutrient composition (typically < 800 calories/day), they are as a group effective in weight loss and metabolic improvement^41^ while typically maintaining fat‐free mass.^107^, ^108^, ^109^ VLCD with carbohydrate levels of <50 g/day result in nutritional ketosis. To demonstrate compliance with the dietary plan and optimize weight loss, BrAce can be monitored. In fact, measurement of BrAce has been shown to correlate with weight loss and adherence to the prescribed VLCD intervention.^110^

After bariatric surgery, patients are monitored by a multidisciplinary team of physicians, nurses, and dietitians, whose main objective is to ensure that patients maintain optimal benefits with minimal harm, including that weight loss be predominantly from fat mass components, and the sparing of fat‐free mass. While many patients do achieve these healthy weight‐loss objectives, there is a minority who, for a variety of reasons, may be unable to adhere to nutritional recommendations and suffer the consequences of poor oral intake if not rescued in a timely manner. It is imperative, therefore, that patients at risk should have their fluid status, electrolyte levels, acid–base balance, and nutritional parameters closely monitored. Comprehensive monitoring may benefit from quantitative measurement of ketone bodies (e.g., BrAce), which could be used to enhance post‐bariatric diabetes management and distinguish between types and degrees of ketoacidosis such as SGLT2‐associated euglycemic ketoacidosis and post‐surgery starvation ketoacidosis.^111^, ^112^

Starvation ketoacidosis can result secondary to poor oral intake following bariatric surgery.^113^ Poor oral intake rapidly depletes hepatic and muscular glycogen stores. Aggravating this situation is the vomiting and dehydration that are occasionally present and stimulate the sympathetic system resulting in released cortisol, adrenaline, glucagon, and growth hormone, which in turn suppress insulin secretion. The cumulative effects of these changes are lipolysis and FFA production from adipose tissue and hepatic ketone formation, the basis for starvation ketoacidosis.^114^ Therefore, all patients who present with severe acid–base imbalance should undergo ketone body measurement since their elevation is a key determinant that ketoacidosis is the underlying mechanism for metabolic acidosis.

In clinical settings, it is important to differentiate starvation ketoacidosis from DKA, since the treatment for each condition can have a critical impact on the outcome of these emergency conditions. DKA is a potentially lethal condition that is more common in patients with type 1 diabetes who exhibit poor compliance or inadequate insulin replacement therapy. It is usually associated with hyperglycemia and dehydration, and is typically managed with dedicated protocols that call for aggressive rehydration and insulin administration in an attempt to correct the acid–base imbalance. It is important to distinguish diabetic from starvation ketoacidosis since glucose administration can be a life‐saving measure in the latter situation. Although DKA after bariatric surgery is an uncommon event, it has been documented in patients with type 1 diabetes who have undergone gastric bypass.^112^ Anesthesia and surgical stress, abrupt discontinuation of insulin or inadequate treatment in the perioperative period, postoperative infection, prolonged poor oral intake, and severe dehydration can be the precipitating causes for postoperative DKA.^115^

## 4 SUMMARY

The measurement of ketone body concentration (BOHB or acetone) can provide valuable information. Diseases such as congestive heart failure, ketoacidosis, and genetic disorders create an elevated ketosis which, in many cases, correlates to disease severity. Because the magnitude of the increased ketosis is typically related to disease severity, early detection can aid in modifying behaviors before disease symptoms clinically manifest. The intentional induction of elevated BOHB concentrations can be used to treat obesity and obesity‐associated diseases such as brain disorders and type 2 diabetes. Elevated BOHB levels provide a benefit through modification of mitochondrial energy production and through reduction of insulin and blood glucose when achieved via glucose restriction. Measurement of ketosis is critical to verify that a therapeutic level of ketosis has been achieved and maintained. Frequent measurement of ketosis can allow users to adjust and personalize their diet and behaviors to maintain therapeutic levels of ketosis. A case study of bariatric surgery demonstrates that monitoring ketone bodies before and after surgery may optimize surgical outcomes and reduce complications.

To date, monitoring ketone concentrations has been shown to address three conditions: ketoacidosis (prevention, acidosis severity, and resolution monitoring), improvement of type 2 diabetes (achieve nutritional ketosis and dietary adherence), and epilepsy (optimize seizure control). These methods of ketone monitoring can be used as starting points for the other conditions reviewed. Additional studies are needed to demonstrate the value of ketone body monitoring for the other diseases that generate elevated ketosis. For therapeutic ketosis, ketone monitoring is necessary, at a minimum, to demonstrate a therapeutic dose has been achieved. Additional research is needed to better quantify the therapeutic dose as a function of subject‐specific factors, which include disease severity, demographics, and genetics.

## CONFLICTS OF INTEREST

Joseph C. Anderson consults for and holds stock in Medamonitor. Samer G. Mattar has no conflicts to report. Frank L. Greenway​ reports serving on science advisory boards for JC USA, Regeneron Pharmaceuticals, and Pfizer; consulting for Jazz Pharmaceuticals, Basic Research, Dr. Reddy's Laboratories, General Nutrition Corporation, Melior Discovery Inc., Novmeta Pharma, Novo Nordisk; grants from Melior Discoveries, Novmeta Pharma; and stock/stock options from Ketogenic Health Systems, Inc., Plensat Inc., UR Labs. Richard J. Lindquist consults for Medamonitor.
